# The effect of long-term brine discharge from desalination plants on benthic foraminifera

**DOI:** 10.1371/journal.pone.0227589

**Published:** 2020-01-14

**Authors:** Chen Kenigsberg, Sigal Abramovich, Orit Hyams-Kaphzan

**Affiliations:** 1 Department of Geology and Environmental Sciences, Ben-Gurion University of the Negev, Beer-Sheva, Israel; 2 Geological Survey of Israel, Jerusalem, Israel; Universitat Bremen, GERMANY

## Abstract

Desalination plants along the Mediterranean Israeli coastline currently provide ~587 million m^3^ drinking water/year, and their production is planned to increase gradually. Production of drinking water is accompanied by a nearly equivalent volume of brine discharge with a salinity of ~80 that is twice the normal, which can potentially impact marine ecosystems. The goal of this study was to examine whether benthic foraminifera, a known sensitive marine bio-indicator, are affected by this brine-discharge. For that, we investigated the seasonal and cumulative effect of brine discharges of three operating desalination facilities along the Israeli coast. Those facilities are located in Ashkelon, Hadera, and Sorek. The brine-discharge in the first two desalination plants is associated with thermal pollution, while the Sorek facility entails increased salinity but no thermal pollution. In four seasonal cruises during one year, we collected surface sediment samples in triplicates by grabs from the outfall (near the discharge site), and from a non-impacted control station adjacent to each study site. Our results highlight that the most robust responses were observed at two out of three desalination shallow sites (Ashkelon and Hadera), where the brine was discharged directly from a coastal outfall and was accompanied with thermal pollution from the nearby power plants. The total foraminiferal abundance and diversity were, generally, lower near the outfalls, and increased towards the control stations. Moreover, changes in the relative abundances of selected species indicate their sensitivity to the brine discharge. The most noticeable response to exclusively elevated salinity was detected at Sorek discharge site, where we observed a sharp decline in organic-cemented agglutinated benthic foraminifera, suggesting that these are particularly sensitive to elevated salinity. The herein study contribute new insights into the effect of brine discharge from desalination plants, on benthic foraminifera, and propose a scientifically-based ecological monitoring tool that can help stakeholders.

## Introduction

**Fig 1:**
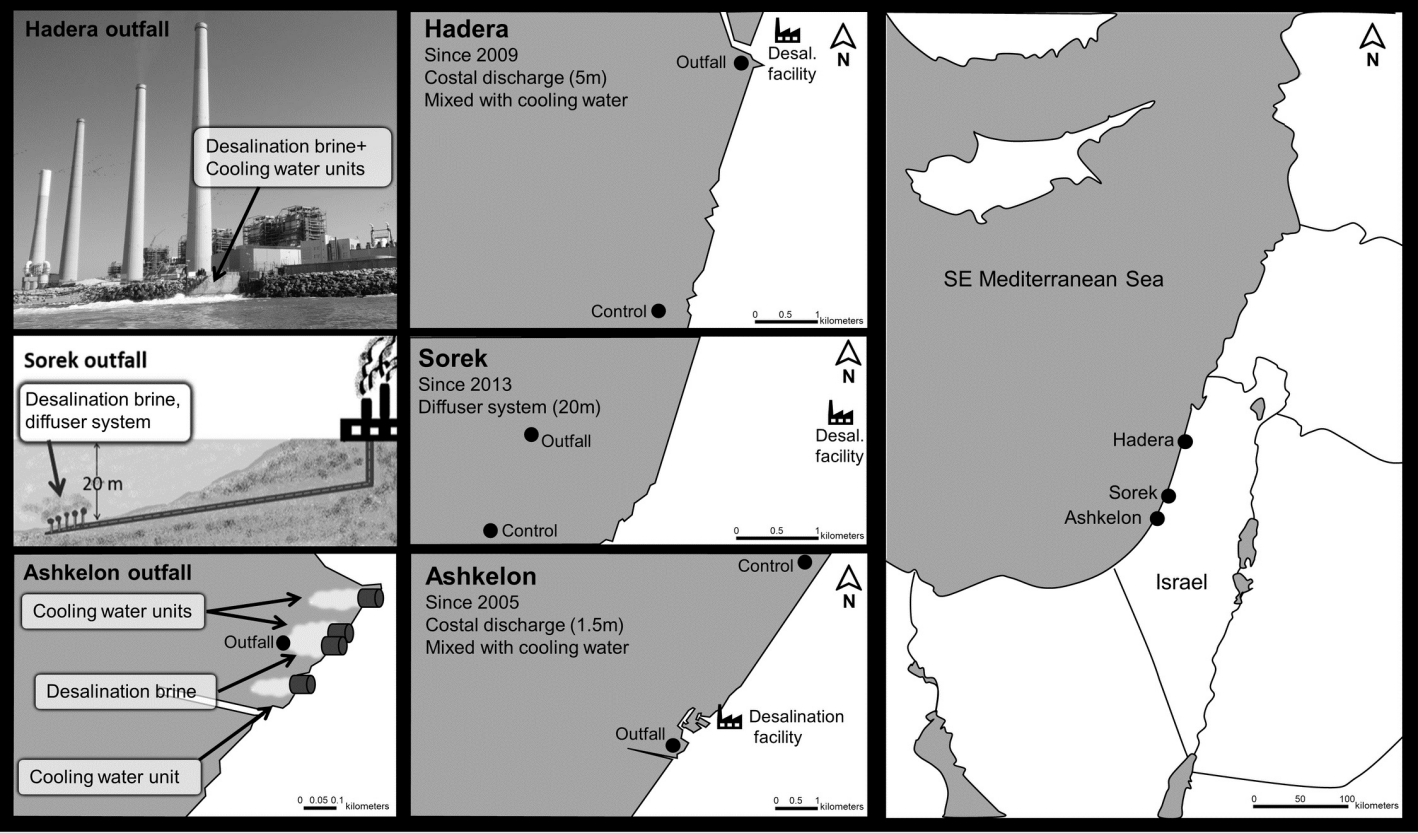
Research area. A schematic map indicating the locations of the Hadera, Sorek, and Ashkelon desalination facilities (top, middle and lower images, respectively) along the southeastern Mediterranean Israeli coastline. The locations of the outfall and control stations at each desalination facility are shown with circles (specific locations are detailed in Table 1). The distance between the Hadera and Sorek desalination facilities is ~60 km, while the distance between Sorek and Ashkelon is ~40 km. The picture was taken by C. Kenigsberg.

Population growth, rising standards of living, industrial proliferation, water supply contamination and climate change are leading to water shortages worldwide. With the advance of technology and reductions in production costs, seawater desalination is becoming the preferred choice for meeting the increasing demand for water and producing a reliable supply. Seawater reverse osmosis (SWRO) is one of the most commonly used desalination technologies [1,2], including in Israel, where it is predicted to be more commonly used in years to come [3]. Currently, five large-scale SWRO desalination facilities are operating along the relatively short (~100 km) Israeli coastline along the southeastern Mediterranean Sea. These facilities are located in Ashkelon (initial operation year: 2005), Palmahim (2007), Hadera (2009), Sorek (2013), and Ashdod (2015) and currently produce ~587 million m^3^ y^-1^ of freshwater [4] (Fig 1). Discharge of the brine effluent to the coastal environment is achieved either by direct surface release along the coastline, as is the case at Hadera and Ashkelon, or through a diffuser system away from the shore, as is the case at Sorek [5,6]. To maximize dilution, the brine is usually mixed with cooling water from adjacent power plants. The result is a warm (~25% over ambient temperature) saline (up to 10% over ambient salinity) floating brine plume that can be found up to a few kilometers away from the discharge site [6,7].

**Table 1:** The locations of the outfall and control stations at each one of the three desalination facilities: Ashkelon, Hadera, and Sorek.

Desalination facility	Site	Location		Bottom water depth (m)
		Lat. (N)	Lon. (E)	
**Ashkelon**	Outfall	31.3796	34.3102	1.5
	Control	31.4067	34.3283	4
**Sorek**	Outfall	31.5652	34.4123	20
	Control	31.5555	34.4077	20
**Hadera**	Outfall	32.2793	34.5294	5
	Control	32.2497	34.5208	6

The growing use of desalinated water has highlighted the close interactions between large-scale facilities and their influence on the environment [5,8], where key issues (among others) include land use, groundwater contamination, energy demand, and noise [9,10].

The current efficiency of SWRO is ~40–50%; thus, the brine waste that is generated and discharged into the sea has approximately twice the salinity of the ambient seawater, which is up to ~40 on the Israeli shelf. Furthermore, the discharge of concentrated brine (approximately 70–80), and chemicals (e.g., phosphonates and ferric sulfate-based coagulants) that are added in the desalination process [5,8] and released to the surrounding coastal zone has become an environmental issue of the highest concern. The concentrated brine is denser than ambient seawater and therefore sinks and flows along the sea bottom, just above the benthic environment [11,12]. Given all of the above, the assessment of brine propagation and its complex interactions with regional dynamics, ambient populations, and marine ecosystems is necessary. These are especially required for emphasizing the effects on the benthic fauna inhabiting shallow sandy sediments off the Israeli coast. This environment is more prone to be affected because of wave-induced longshore currents together with its oligotrophic nature, particularly since the damming of the Nile River [13]. To date, the short (seasonal)- and long-term (years) effects of this brine discharge have been poorly studied.

### Benthic foraminifera

Benthic foraminifera represent ideal model organisms for studying the impact of salinity on coastal ecosystems. These unicellular heterotrophic organisms are considered to be highly sensitive to marine perturbations and are widely used to monitor marine pollution, e.g. [14–19]. Most of the studies on salinity effects on benthic foraminifera have focused on their exposure to low salinities due to freshwater influxes, e.g. [20,21], in which it has been shown that foraminifera responds via changes in numerical abundance and diversity as well as the assemblage composition. However, only a few studies have documented the effects of elevated salinity on benthic foraminifera. For example, in cases of extreme salinity levels, the shells of cosmopolitan species such as *Ammonia tepida* are prone to deformation and abnormalities [16,22]. More previous studies on hypersaline environments, such as the Salwa Bay (Saudi Arabia) [23], and the Abu Dhabi Lagoon, Persian Gulf [24,25] reported on relatively low diversity of benthic foraminiferal assemblages. In Israel, Arieli et al. in 2011 [26] and later Titelboim et al., in 2016 [27] studied the effect of thermal pollution on shallow-water benthic foraminifera inhabiting a unique hard-bottom environment near the Hadera power plant before and after the establishment of the desalination plant. Their results indicated that elevated temperatures, not salinity, are the most significant environmental stressors affecting the local foraminiferal community. Furthermore, they showed that many of the shallow-water species of the eastern Mediterranean (some of which are known as Lessepsian migrators) are preadapted to high salinity. However, the mixing of warm cooling water at Hadera causes only a modest elevation of salinity above the typical ambient background. Hence, it is essential to explore the possible role of elevated salinity as a stressor on benthic foraminifera near desalination plants that are not associated with power plants. Moreover, there is still a lack of knowledge regarding the effect of brine from desalination plants on the ecosystems of soft-bottom sediments, which represent the main ecological domain of the Mediterranean shelf.

The herein study presents the results of one year of monitoring at three desalination sites, thus showing the long-term effects of continuous brine discharge on benthic foraminifera in soft sandy habitats as representatives of the eukaryotic benthic community. It is a part of a large-scale project that aimed to evaluate the possible spatiotemporal effects of concentrated brine discharges along the whole Israeli coastline. The other parts of that project described the effects on benthic heterotrophic bacteria [28] and on marine food webs [29].

### The three desalination facilities

#### Ashkelon facility

The Ashkelon desalination facility began operating in 2005 with the production of desalinated water at a rate of 115 M m^3^ y^-1^, and it is an adjunct to a power plant that has been adding cooling water to the seawater for the last 30 years. The desalination brine and the cooling water are discharged to the sea at approximately the same point but from separate open-coastal channels (Fig 1). Published reports indicate that due to the mixing of the brine with the cooling water after discharge, the salinity and temperature create a disturbed area that extends approximately 1.2 km^2^ to the west and south showing higher temperatures (difference of 0.5–5°C relative to the background) and salinity values (40.46–42.99, increase of 2.5%-9% relative to the background, respectively) near the seabed [30,31].

#### Hadera facility

The Hadera desalination facility began operating in 2009, producing desalinated water at a rate of 127 M m^3^ y^-1^. Here, the brine is associated with a preexisting power plant that has operated since the early 1980s; however, mixing with cooling water is performed before its discharge into the sea via an open-coastal channel at this site (Fig 1). Published reports show that the disturbed area extends approximately 0.9 km^2^ to the south and west, showing higher temperatures (difference of 1–2°C relative to the background) and salinity values (40.71–41.96, increase of 2.5%-6% relative to the background, respectively) near the seabed [32].

#### Sorek facility

The Sorek desalination facility began operating in 2013 and is not associated with any power plant. In contrast to Hadera and Ashkelon, the brine is discharged through a diffuser system at a 20 m water depth at this facility [33]. Published reports show that the brine spreads between 0.5 and 1.5 km^2^ to the north and southwest, showing higher salinity values (39.63–43.52 increased by 1%-9.7% relative to the background, respectfully) [33].

## Experimental design and methods

### Sampling locations

The material examined in this study was collected during four cruises conducted from 2016–17 aboard the R.V Mediterranean Explorer in proximity to three large-scale SWRO desalination facilities: Ashkelon, Sorek and Hadera, from south to north (Fig 1). To track the seasonal pattern at each site, sediment samples were collected during June 2016 (summer), November 2016 (fall), January 2017 (winter), and April 2017 (spring), in addition to water column measurements. There was no need for sampling permission in that area because it is not located within a marine reserve. The field studies included only foraminifera and did not involve endangered or protected species.

This study is based on the sampling of two locations at each site: an outfall station in close proximity to the brine outfall and a control station at the same depth located several kilometers away from the discharge point (Fig 1 and Table 1). The control stations were not influenced by the physicochemical characteristics of brine; i.e., the water temperature and salinity were similar to the ambient values. Water temperature and salinity profiles were measured for each site in real-time by an MS5 probe (Hydrolab MS5 water quality multiprobe, USA), focusing on the values measured at the bottom of the water column at the outfall and control stations. Triplicate surface sediment samples of the top 1 cm of the sediments were collected via a Van-Veen grab for further analyses of grain size, total organic carbon (TOC), trace and major elements, and benthic foraminiferal assemblage composition. In total, 72 sediment samples were sampled from June 2016 to April 2017.

### Sediment analyses

The sediment total organic carbon (TOC) weight percentage was measured via potassium dichromate (4.0 N K_2_Cr_2_O_7_) digestion and potentiometric titration with (2.0 N) Fe(NH_4_)_2_(SO_4_)_2_6H_2_O according to a previous report [34], with a detection threshold of 0.02%. Grain size analysis was conducted using Malvern MS-2000 laser diffraction over the particle size range of 0.02 to 2000 μm. The measurement procedure included dispersion (using sodium hexametaphosphate solution), stirring for 5 min, and ultrasonication for 30 s. The raw laser diffraction values were transformed to a particle size distribution using the Mie scattering model, with optical parameters of RI = 1.52 and A = 0.1. For chemical composition determination, 24 subsediment samples were fully dissolved. Major and minor element (Si, Al, Fe, Ti, Ca, Mg, Na, K, P, S, and Sr) concentrations were determined by inductively coupled plasma—optical emission spectrometry (ICP-OES; Optima 3300, Perkin-Elmer) following lithium metaborate fusion in platinum crucibles e.g. [35], and trace metal (As, Ba, Be, Cd, Cr, Cu, Mn, Ni, Pb, Sn, Se, and Zn) concentrations were determined by inductively coupled plasma-mass spectrometry (ICP-MS; NexION, Perkin-Elmer) after being dissolved by sodium peroxide sintering in zirconium crucibles e.g. [36]. Standard reference materials were processed and analyzed along with the samples for quality control. The data are better than ±10% for traces and ±5% for majors.

### Foraminiferal analyses

For foraminiferal analyses, the first cm from each grab was collected with a short Perspex-core (triplicates of 50 cm^3^/sample) and immediately preserved in 95% ethanol on board. In the laboratory, the samples were stained with Rose bengal solution (2 g L^-1^ ethanol 95%) for two weeks, later sieved at 63 μm, dried at 50°C. Proximately 100 gram (total sample weight) was subsequently treated with heavy fluids (zinc bromide, ZnBr_2_), at a density of 2.3 gr ml ^-1^, to separate the abundant silicate minerals from the foraminiferal fraction similar to [37] with an exception of the later using Sodium Polytungstate. The remaining sediment was dried, and the 63–2000 μm fraction was used for picking all red-colored foraminifera in the sample, for achieving the live foraminiferal assemblages at the sampling time. Identifications were performed to the most specifically possible taxonomic level (99.4% to the species level) based on [38–44]. Foraminifera were counted in each sample to determine ecological indices such as total and relative abundance and species diversity (S1 Table). Those indices were used to describe the assemblage composition and its characteristics. Numerical abundance was calculated for each sample as the total number of individuals per 1 gram of dry sediment. The same procedure was applied for each species to obtain the assemblage composition.

### Statistical analyses

Statistical analyses were performed using Primer v.6 from the Plymouth Marine Laboratory [45–47] and STATISTICA 10 software. Species numerical abundances were standardized by logarithmic transformation (log(x+1)) to reduce the influence of dominant species. This transformation is usually applied to all entries in the assemblage matrix of counts, biomass, percent cover, etc. [45,47]. Environmental data were normalized and logarithmic transformed (log(x+1)). These steps are typical of a suite of physico-chemical or ecotoxicological variables, which are not on comparable measurement scales. [45,46].

Analysis of similarities (ANOSIM) was applied to the foraminiferal relative abundances to detect significant differences between sites and stations. Principal component analysis (PCA) was applied to the environmental data to detect differences between localities and to identify their contribution to the differences among the different stations. Nonmetric multidimensional scaling (nMDS) was used based on the Bray-Curtis similarity matrix to project the community assemblage patterns in a two-dimensional ordination plot. Points that are close together represent samples that are similar in community composition (or environmental variables, biomarker responses, etc.) and points that far apart correspond to very different values of the variable set. Stress <0.2 indicates a potentially useful two-dimensional representation [46]. Analysis of variance (two-way ANOVA) was performed on foraminiferal abundance and species richness to identify the differences between stations at each site. Also, one-way ANOVA was performed on environmental data to detect differences in salinity between the outfall to control for each site. For this purpose, the seasons were used as replicas. In a case that the ANOVA assumption was violated (not normally distributed and not presenting homogeneity variances), a non-parametric Mann-Whitney U test was performed instead.

## Results

### Environmental conditions

#### Temperature and salinity anomalies

**Fig 2:**
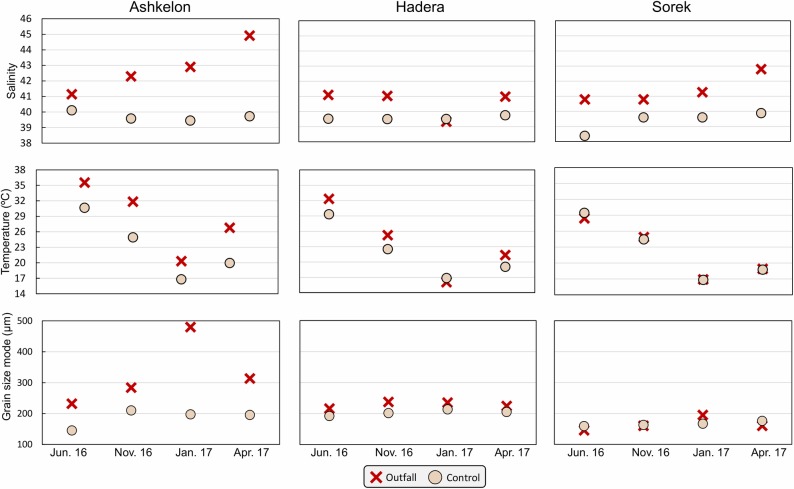
Seasonal environmental measurements. Bottom-water salinities, temperatures, and the grain size mode at each desalination facility.

The expected salinity and/or temperature anomalies between the outfall and control stations were observable at all sites (Fig 2). The salinity was significantly different between the three sites and between the sites outfalls and their respective control stations [F_(2,18)_ = 3.84, p = 0.04 in one-way ANOVA; F_(1,18)_ = 32.91, p<0.001 in one-way ANOVA for salinity difference between outfall and control] (Tables A and B in S2 Tables). The strength and persistence of the anomalies can be evaluated based on a comparison between the control and the outfall stations at each site during the four seasonal sampling campaigns.

#### Ashkelon facility

At this site, the salinity values at the outfall varied between 41 and 45 in the summer and spring, respectively, which was 1 to 5 units higher than the values at the control station (Fig 2 and Table 2). This site also exhibited the highest salinity values compared with the two other sites during spring, winter, and fall. The temperature presented clear seasonal changes, varying between 35.5°C in summer and 20.3°C in winter in the outfall, where the temperature was 7.4°C higher than at the control station (Fig 2 and Table 2). The salinity was different between the outfall and the control stations [F_(1,6)_ = 14.95, p = 0.008 in one-way ANOVA for salinity difference between outfall and control] (Table C in S2 Tables).

**Table 2:** Environmental parameters measurements.

Facility	Sampling season	Temp. (°C)		Salinity		GS mode (μm)		Mn (ppm)		Cr (ppm)		Fe_2_O_3_ (wt.%)	
		Out.	Con.	Out.	Con.	Out.	Con.	Out.	Con.	Out.	Con.	Out.	Con.
**Ash.**	**Summer**	35.5	30.6	41.15	40.1	232.1	154.5	71	253	10	40	0.3	1.3
	**Fall**	31.8	24.9	42.3	39.6	284.2	210.3	50	181	8	42	0.2	0.7
	**Winter**	20.3	16.8	42.9	39.45	480.2	197.6	50	209	10	48	0	1.08
	**Spring**	26.8	19.9	44.9	39.7	314.0	195.6	50	257	6	41	0.24	0.9
**So.**	**Summer**	28.4	29.45	40.8	38.4	145.6	159.4	580	403	240	93	2.8	1.9
	**Fall**	24.9	24.4	40.8	39.6	160.4	163.0	341	346	68	118	1.7	0.3
	**Winter**	16.9	16.8	41.2	39.6	195.2	167.1	766	368	185	83	2.5	1.89
	**Spring**	18.8	18.7	42.8	39.8	160.5	176.0	934	396	234	85	3.71	1.39
**Had.**	**Summer**	32.3	29.3	41.1	39.52	216.4	192.1	73	112	8	13	0.3	0.4
	**Fall**	25.2	22.5	41	39.5	237.7	201.7	77	100	10	14	1.9	0.4
	**Winter**	16.05	16.91	39.3	39.5	235.6	213.7	99	139	14	6.9	0.18	0.47
	**Spring**	21.3	19	40.9	39.7	224.5	205.3	62	88	5	8	0.26	0.26

Seasonal bottom-water temperatures and salinities and sediment analyses (grain size, TOC, Mn, Cr, Fe_2_O_3_) at the outfall (Out.) and the control (Con.) stations of the three desalination facilities.

#### Hadera facility

At the Hadera outfall station, the salinity values varied from ~41 to ~39 from summer to winter, respectively (Fig 2). A moderate salinity anomaly up to 1.5 was noted between the outfall and the control stations. The only exception was detected during winter when the values at the two stations were similar. The temperature recorded at both stations presented clear seasonal changes and varied between 32°C and 16°C at the outfall station and between ~29°C and ~17°C at the control station during summer and winter, respectively. Therefore, a maximum anomaly of 3°C was recorded at the Hadera site during summer (Fig 2 and Table 2). The salinity of the outfall in Hadera was comparable to that of its control stations [U = 4, n_1_ = n_2_ = 4, p = 0.31 in Mann-Whitney for salinity difference between outfall and control] (Table D in S2 Tables).

#### Sorek facility

At the Sorek site, salinity was the only expected anomaly since this site is not associated with a power plant. At the outfall station, the maximum salinity values were recorded during spring (~43) and were ~3 units above the values at the control station measured at the same time. Temperature records were similar between the outfall and the control stations and varied between ~29°C and ~17°C during summer and winter, respectively (Fig 2 and Table 2). The salinity was different between the outfall and the control stations [F_(1,6)_ = 12.95, p = 0.01 in one-way ANOVA for salinity difference between outfall and control] (Table E in S2 Tables).

### Sediments

#### Grain size

A well-distinguished difference in surface grain size mode (μm) between the three sampling sites was correlated with water depth, as reported for the Israeli shelf [13,48]. An apparent increase in grain size was observed between the deeper Sorek site (20 m) and the shallower Hadera and Ashkelon sites (1.5–6 m; Fig 2). A prominent grain size anomaly between the control and outfall stations was observed only at the Ashkelon site. This phenomenon was partially due to a slight difference in water depth between the outfall and the control stations, which were located at depths of 1.5 m and 5 m, respectively, although both were within the shallow sandy belt. A clear seasonal pattern was observed at this site, mainly at the outfall station, where the grain size mode varied from 232 μm during summer to an exceptionally coarse grain size of 480.2 μm during winter (Table 2 and Fig 2). This sorting of the sediments was probably caused by the strong currents persisting at the shallowest depth (1.5 m) due to the cooling water and desalination water discharged through a pipe. At the Hadera site, the grain size mode values at the outfall station were only slightly higher than those at the control station (up to 30 μm). At the Sorek site, the grain size was homogenous between the stations throughout the year and was finer due to its deeper location (~180 μm; Fig 2 and Table 2). These grain size measurements are similar to those reported by Sivan and Almogi-Labin in 1999 [48], showing a homogenous siliciclastic sandy belt on the shallow Israeli shelf.

#### TOC

Values recorded at the surface sediments of the three sites were very low and homogeneous for all sampling sites and stations (<0.1%). These values are typical of shallow siliciclastic sandy sediments in an oligotrophic sea [48,49], while no influence of extra organic pollution was detected on our sampling dates. Moreover, the damming of the Nile River, the major sediment supplier to the Israeli coast, in 1964 greatly reduced the fine sediments and accompanying nutrients that used to arrive with the Nile flooding cycles every summer [50,51].

#### Trace elements

Most of the measured trace elements showed very low values and did not vary between the sampling stations. The only exceptions were chromium (Cr) and manganese (Mn), which presented slightly higher concentrations at the Sorek outfall station compared to the control station, while an opposite trend was observed at both the Ashkelon and Hadera sites (Table 2).

Most major elements also exhibited very low concentrations with no variation between the sites of the facilities and their corresponding stations. Moreover, the concentrations of the desalination-associated elements P_2_O_5_ and SO_3_ are consistently low at all sites and stations (≤0.1% and <1%, respectively). The only exception was the concentration of Fe_2_O_3_, which exhibited higher values at the outfall station at the Sorek site and the control station at the Ashkelon site. The concentration of Fe_2_O_3_ at the Hadera site was low at both stations compared to that at the two other facilities (Table 2).

An ANOSIM test was performed to evaluate the differences between all three sites and six stations, regarding all environmental parameters (salinity, temperature, grain size, Cr, Mn, and Fe_2_O_3_). The results indicate that there is a difference between the sites and the stations (sites: R = 0.501, p = 0.002%; stations: R = 0.378, p = 0.4%).

### Benthic foraminiferal assemblages

**Fig 3:**
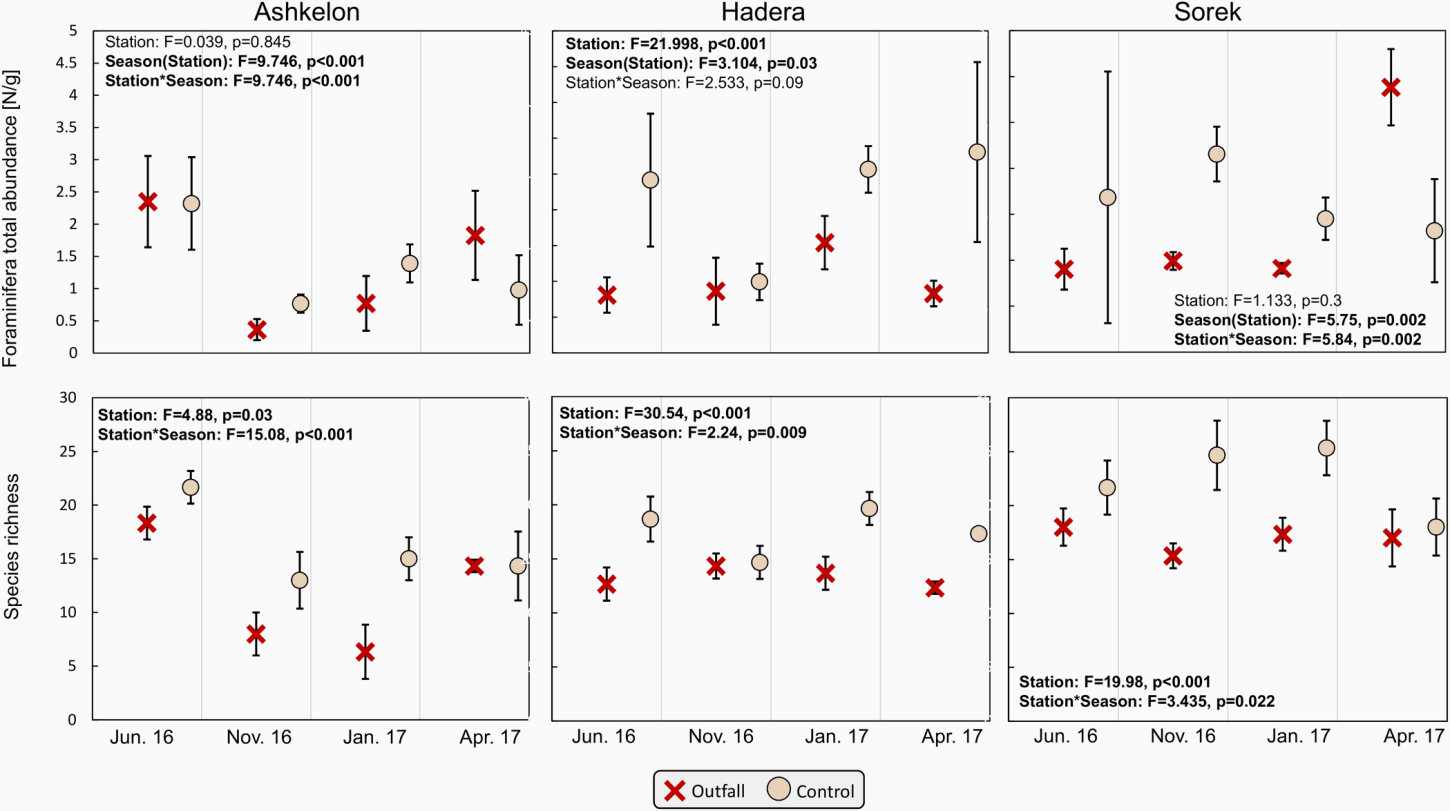
Abundance richness indexes. Foraminiferal total abundance (top) from June 2016-April 2017, calculated from the number of individuals per sample (N) divided by the sample's dry weight (grams). Species richness (bottom), calculated by the number of species in each sample. The X and circles represent the averages of the outfall and control respectably. Solid lines represent the standard deviation of triplicate samples. Two-way ANOVA was performed for each index at each site; the results (statistic F and probability P-value) are presented on each diagram. Significant differences are presented in **bold**.

The total live foraminiferal abundances recorded at all studied sites were low, varying from 0.2 to a maximum of 4.5 individuals per gram dry sediment [N/g] (Fig 3). The lowest numerical abundance and species richness were found at the shallowest site of Ashkelon, and the highest values were found in the deepest site of Sorek. These results were also supported by the equitability indices (J'): for the Ashkelon site, J' = 0.63; for Hadera, J' = 0.66; and for Sorek, J' = 0.77. In addition, it was clearly shown that at all three sites, species richness was higher or at least similar at the control stations with respect to the outfall stations and changed on a seasonal basis (Fig 3). The results can indicate that tolerance of benthic foraminifera to anthropogenic brine is species-dependent and that many species are sensitive to elevated salinity.

The overall species richness at all sites varied seasonally between 5 and 28 species. The highest numbers were recorded at the control station of the deeper water site of Sorek during fall 2016 and winter 2017 (Fig 3). This site was generally characterized by higher species richness values compared with the two shallower sites, Hadera and Ashkelon.

#### Species composition

**Fig 4:**
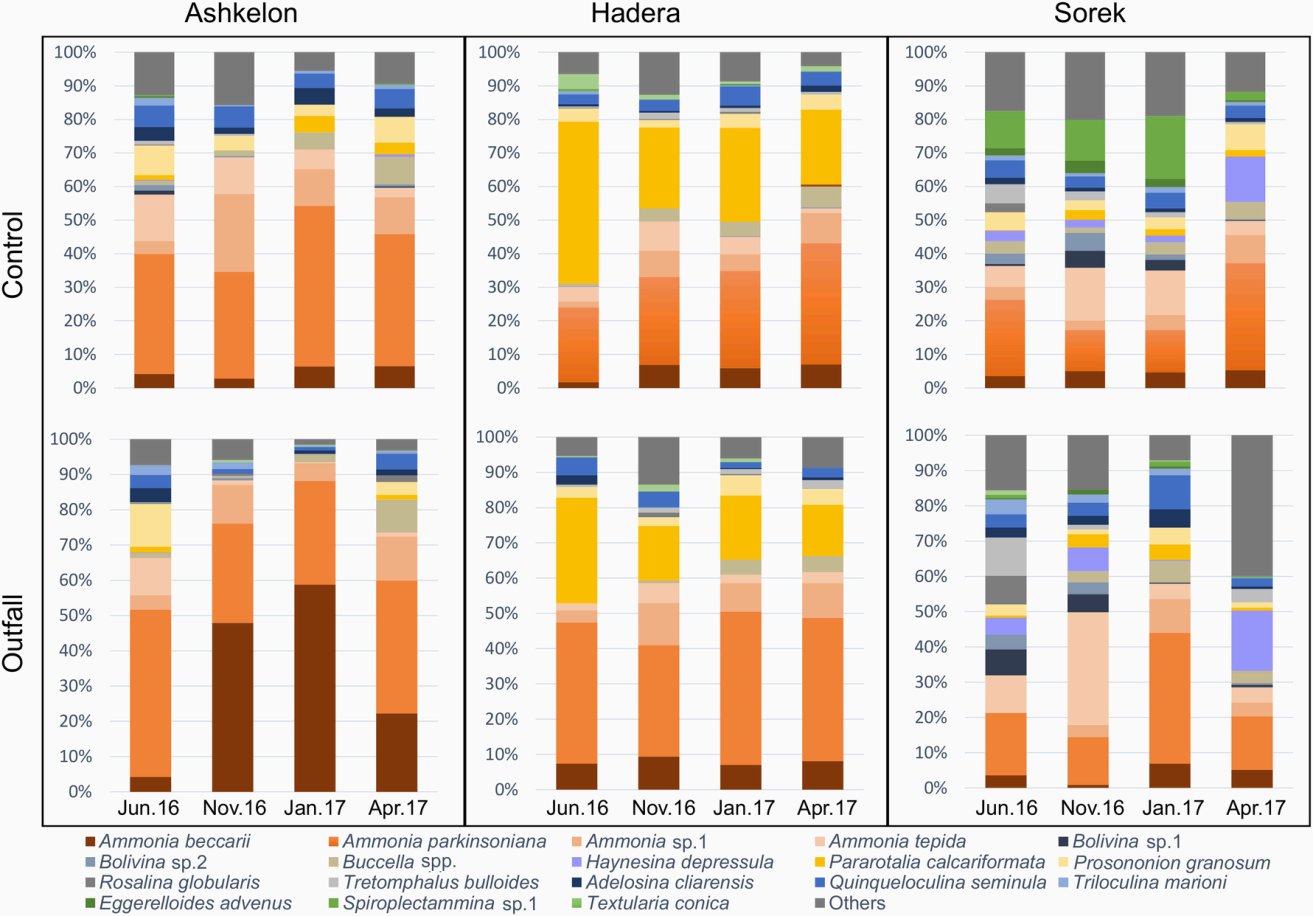
Cumulative relative abundances (%). Represented by the averages from the triplicate of the most common species (>5% for at least one station) at the control stations (top) and the outfall stations (bottom), divided into three sampling sites: Ashkelon, Hadera, and Sorek. Four sampling periods: June 2016 (summer), November 16 (fall), January 17 (winter), and April 17 (spring).

*Ammonia parkinsoniana* was by far the most dominant species, typically constituting up to 60% (an average of 45% ±32%) of the total assemblage at shallow water depths from 3 to 9 m at Ashkelon and Hadera. Other common species at these water depths were *Ammonia beccarii* (avg. 11.5% ±12%), *Ammonia* sp.1 (10% ±7%), *Ammonia tepida* (11% ±11%), *Pararotalia calcariformata* (17% ±29% mainly in Hadera), *Buccella granulata* (5.5% ±5%), and *Spiroplectammina* sp.1 (10% ±14% only in Sorek), each constituting up to 20% of the total assemblage. (Fig 4 and S1 Table).

A clear bathymetric zonation based on foraminiferal species was observed in this study, as reported in previous studies [13,52]. For example, *A*. *tepida* replaced *A*. *parkinsoniana* and *A*. *beccarii* at the deeper site of Sorek compared with the other two shallower sites (Hadera and Ashkelon). On the other hand, the relatively high abundance of stained organic-cemented agglutinated species (arenaceous foraminifera) such as *Eggerelloides advenus* and *Spiroplectammina* sp. 1 at the control station of the Sorek site suggests that these species inhabit shallower depths than previously reported [13,53,54]. The Lessepsian invader species, *P*. *calcariformata* that was mostly found in the Hadera site seemed to be restricted to the northern and central coast of Israel, where it was abundant in both hard-bottom and soft-sediment habitats and frequently accompanied with high water temperatures [27,55].

### Statistical analyses

**Fig 5:**
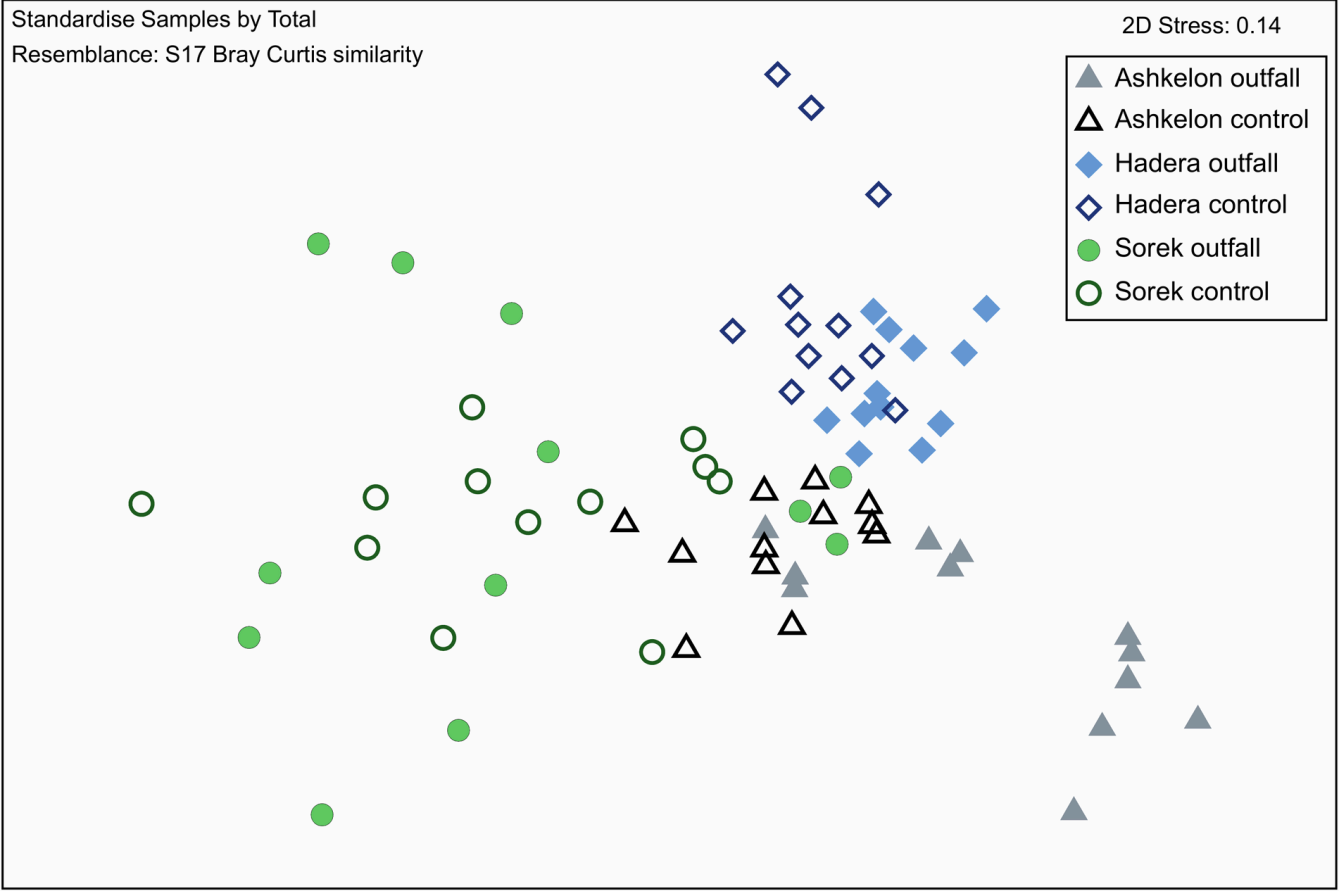
Nonmetric multidimensional scaling (nMDS) based on foraminiferal relative abundance. The nMDS is showing similarity distances between the three sites and stations. Each mark represents one sample. All replicates and sampling seasons are shown. Stress <0.2 results in a potentially useful 2-dimensional picture [46].

In order to illustrate the visual statistical differences between all sampling sites and stations, we performed an nMDS plot diagram using foraminiferal relative abundance. This diagram showed a clear separation between the three sampling sites (Ashkelon, Sorek, and Hadera) and stations (outfall vs. control) (Fig 5). ANOSIM test was done to verify the visual separation of the nMDS with statistical values. It confirmed that the foraminiferal assemblages of the three sites were different, although Ashkelon was more similar to Hadera than both sites were to Sorek (**Ashkelon, Hadera:** R = 0.685, p = 0.001%, **Ashkelon, Sorek:** R = 0.557, p = 0.001%, **Hadera, Sorek:** R = 0.662, p = 0.001%). This finding means that the foraminiferal assemblages of the three sites are incomparable, potentially to their geographical, physical, chemical, or sedimentological differences (substrate type).

**Fig 6:**
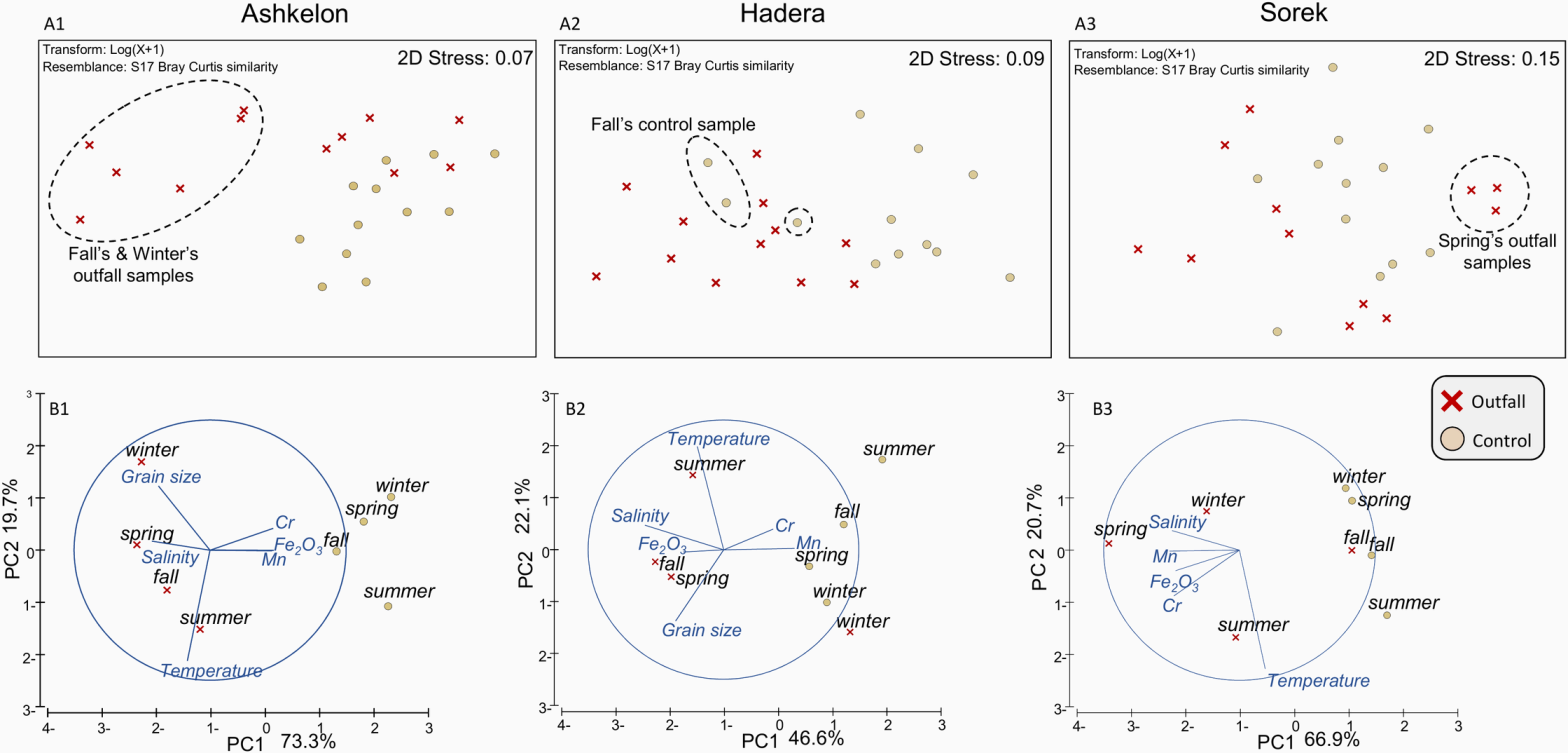
**A1-A3. nMDS plots based on foraminiferal assemblages**. Show the dissimilarity distance matrix between the outfall and control station at each site. Each mark represents one sample. All replicates are shown. Samples with exceptional abundance or species richness values are circled. **B1-B3**. PCA plots of the environmental data. The vector presented only if the correlation is > 0.9. The specific variable values for contribution are detailed in Table 3.

To evaluate the independent influence of the sites based on foraminiferal assemblages, we performed a separate ANOSIM test per each of the three sites. These analyses displayed significant differences between the foraminiferal assemblages of the outfall and control stations (Table 3). These differences are also evident in the nMDS diagram that separates most of the outfall samples from the control samples (Fig 6A). The statistical test indicated that the differences in total abundance between the stations were significant only at Hadera (two-way ANOVA: F _(1,16)_ = 21.998, p<0.001; S1 Fig). However, at the Ashkelon and Sorek sites, the interaction between season and station was significant (two-way ANOVA: at Ashkelon: F_(6,16)_ = 9.75, p<0.001; and Sorek: F_(6,16)_ = 5.84, p = 0.002; Fig 3). The PCA test was applied to the environmental parameters to detect variances between areas and to classify their influence on the differences among the different stations. The main axis (PC1) of the PCA ordination showed differences between stations, indicating that salinity was as effective as the concentrations of Mn, Cr, and Fe_2_O_3_. Desalination outputs (elevated salinity and metals) were effective in opposite directions at Ashkelon and Hadera and the same direction at the Sorek site (Fig 6B and Table 3).

**Table 3:** Results of ANOSIM and PCA.

Analyze		Ashkelon		Hadera		Sorek	
ANOSIM	Differences between outfall and control stations	R = 0.667, p = 0.05		R = 0.676, p = 0.04		R = 0.759, p = 0.02	
PCA	Environmental variables	PC1 (73.3%)	PC2 (19.7%)	PC1 (46.6%)	PC2 (22.1%)	PC1 (66.9%)	PC2 (20.7%)
	Salinity	**0.428**	0.07	**-0.581**	0.189	**-0.495**	0.151
	Temperature (°C)	0.168	**-0.85**	-0.194	**0.797**	0.189	**-0.911**
	Grain size–mode (μm)	0.38	0.495	-0.355	-0.551	——	——
	Mn in sediment (ppm)	**-0.469**	-0.002	**0.525**	0.01	**-0.515**	-0.007
	Cr in sediment (ppm)	**-0.465**	0.169	**0.366**	0.158	**-0.483**	-0.351
	Fe_2_O_3_ (wt.%)	**-0.456**	-0.005	-0.298	-0.018	**-0.47**	-0.157

ANOSIM indicates significant differences between the outfall and control stations for each site. PCA analyses identify each variable’s contribution to the differences in environmental conditions between the stations.

## Discussion

### The effect of elevated salinity and temperatures on foraminiferal assemblages

The numerical abundances of the benthic foraminifera inhabiting the shallow sandy habitats of the Israeli coast are naturally very low and typically consist of 1–3 specimens per gram dry sediment. The reason for this is the high dilution and instability caused by rapidly accumulating siliciclastic (quartz-rich) sandy sediments originating from the Nile River [13,54]. Previous studies conducted on living and recently dead foraminiferal assemblages along the Israeli shelf described a general pattern of low abundances and species richness at water depths of 3–20 m. Large numbers occur from 30–40 m water depths in silty sediment habitats with higher TOC [13,53]. No studies had previously been performed on the living foraminifera inhabiting the very shallow and energetic sandy habitat of the Israeli shelf. Our results demonstrate that both the numerical abundance and species richness documented at the three studied desalination plants support previous studies conducted on the sandy belt. [52]

The low foraminiferal abundances in the naturally unstable and diluted sandy habitats of the Israeli coastline (SE Mediterranean Sea) therefore, present a challenge for the detection of the anthropogenic footprint of elevated salinity and temperature in that area. Species richness should be independent and provides a reliable parameter for assessing the diversity of species, even when low, in soft-sediment habitats. Consequently, it is clear that this comparison between the foraminiferal assemblages of the control stations vs. outfall stations should be focused on changes in their relative abundances and species richness, rather than on numerical abundances.

Considering the results of the statistical analyses (nMDS plots and ANOSIM, Fig 5), it was clear that for evaluating the impact of brine discharge and temperature anomalies on the foraminiferal communities, each site must be analyzed separately, as an independent case study. Therefore, the statistical analyses of comparing the control and outfall stations were based on separate comparisons per each site. An additional parameter separating sites was the metal concentrations. While higher concentrations of metals were recorded at the control stations of the Ashkelon and Hadera sites, at the Sorek site, the higher values were recorded at the outfall station. This might be related to a different sedimentary regime or other pollution sources, which are out of the scope of the herein study. At all sites, seasonal changes in temperatures made the most substantial contribution to differences between stations on-axis PC2 (Fig 6B and Table 3).

### The Ashkelon site

**Fig 7:**
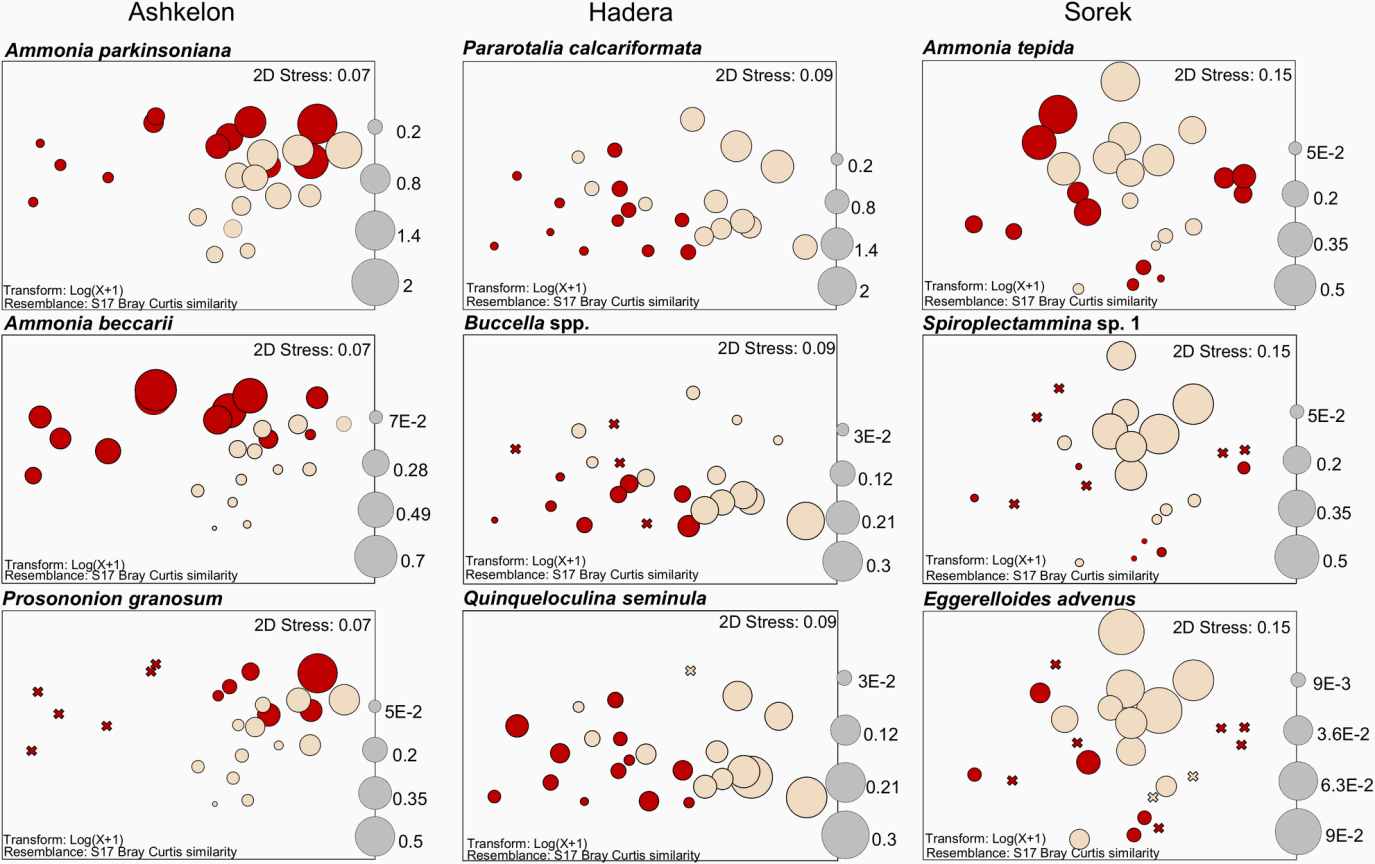
Differences in relative abundances of selected foraminiferal species between the outfall to the control. The nMDS plot shows the dissimilarity distance between stations, with a bobble projection of the abundances of selected foraminiferal species at each desalination facility site: Ashkelon, Hadera, and Sorek from left to right. The size of the circle represents the numerical abundance (N/g) after logarithmic transformation (log(x+1)) of the data. An X indicates the total absence of the species in a sample. Red indicates the outfall station, and beige indicates the control station. Each mark represents one sample. All triplicate samples and seasons are shown.

In this southern region of the Israeli coast, the temporal distribution of brine is determined by the flow rate from the desalination and power plants combined with the velocities of the longshore and coastal currents and natural wind in that region [31]. A possible explanation for the low concentration of desalination-associated metals near the outfall station is the presence of the desalination facility coastal pipeline combined with strong currents and possibly different sedimentary regimes (Fig 6B1). nMDS ordination plots based on log-transformed data of foraminiferal assemblages combined with the ANOSIM and ANOVA statistical tests indicate significant differences between the control and the outfall stations during fall and winter (Figs 3 and 6A1 and Table 3). These differences are expressed in both higher species richness (two-way ANOVA F_(1,22)_ = 4.88, p = 0.03; Figs 3 and S1) and higher relative abundance of *Ammonia parkinsoniana* and *Porosononion granosum* at the control station. *Ammonia beccarii* showed an opposite trend, being highly abundant at the outfall station, possibly because it prefers the coarser sediments characteristic of the energetic shallower water depths (see also [13,56]; Figs 4 and 7 and S1 Table). The seasonal differences between the stations might reflect several parameters: 1. a combined or specific anthropogenic effect of elevated salinity and/or temperature, which might be stronger during winter and fall due to changes in current velocity and strength, and 2. differences in grain size due to the several-meter difference in water depth between the two stations, as stronger currents from the coastal outfall spread fine particles to the surrounding area (as the control station).

### The Hadera site

In this region, the influence of the discharged cooling water jets is mainly observed near the coast; with increasing distance from the coastline, the distribution of sea surface salinity is more affected by the velocities of the winds and natural currents [32]. Providing a similar explanation to that for Ashkelon regarding the lower concentration of trace metals at the outfall station (Fig 6B2). nMDS ordination plots, ANOSIM, and ANOVA also revealed significant differences between the control and outfall stations during all seasons, with one exception during fall (Figs 3 and 6A2 and Table 3), when strong eastern winds were recorded at the time of sampling. These differences are expressed in higher species richness (two-way ANOVA: F_(1,22)_ = 30.54 p<0.001, Figs 3 and S1) and a slightly higher relative abundance of *Pararotalia calcariformata* at the control station (Figs 4 and 7 and S1 Table). On the other hand, the species *Buccella* spp. (mainly *B*. *granulata*) and *Quinqueloculina seminula* did not display any noticeable differences in their relative abundances between the stations, indicating that they are less sensitive to salinity and temperature anomalies (Fig 7 and S1 Table). At this site, the two stations were characterized by similar grain sizes; therefore, the differences in the foraminiferal assemblages were more consistent with an anthropogenic footprint of elevated salinity and temperatures, rather than depth-related changes in sediment grain sizes (the type of substrate) or water energy. According to the reports of the Israel Electric Corporation for the Hadera site, during fall 2016, two of the production units of the electrical power plant did not operate, leading to a decrease in the throughput and spreading of cooling water [31]. This finding suggests that at this site, the elevated temperature has a greater influence on the benthic foraminiferal assemblages than elevated salinity, especially for *P*. *calcariformata*. This interpretation was also reported by Titelboim et al. in 2016 [27].

To conclude, both Ashkelon and Hadera sites consist of similarly structured desalination facilities operating for a long time, from 2005 and 2009 respectively. In addition, both sites are located in a shallow energetic coastline with a sandy substrate. Hence, it is not surprising that both are characterized by a similar poor benthic foraminiferal assemblage.

### The Sorek site

This desalination plant site, located at an ~20 m water depth, has a diffuser system and is not associated with a power plant, meaning that no temperature or grain size anomaly exists between the outfall and the control stations. Therefore, this site represents an ideal case study for evaluation of the possible effect of brine discharge alone (since 2013) on benthic foraminifera, with minimum interference from other natural or anthropogenic factors. Moreover, as the deepest site with finer sediments and lower water energy, this site exhibited the highest species richness (up to 28 species/sample) and therefore provided a better representation of the soft-sediment benthic foraminiferal community (Fig 4). nMDS plots, ANOSIM, and ANOVA showed significant differences between the control and outfall stations (Figs 3 and 6A and Table 3). The only exception was observed during spring sampling, when a large number of juvenile (unclassified) miliolids were recorded at the outfall station, resulting in higher total abundance than that at the control station. These differences are expressed in higher species richness (two-way ANOVA: F_(1,22)_ = 19.98, p<0.001, Figs 3 and S1), with a particularly high relative abundance of the organic-cemented agglutinated species *Spiroplectammina* sp. 1 and *Eggerelloides advenus* observed at the control station (Figs 4 and 7 and S1 Table). Typically, the outfall station was either barren or showed scarce occurrences of organic-cemented agglutinated species, while the control station exhibited over 50 specimens per sample/gram. This observation is similar to the absent of agglutinated foraminifera in the hypersaline Salwa-Bay [23]. The differences between the outfall and the control stations could reflect a separate or synergic anthropogenic effect of elevated salinity and/or Cr and Mn concentrations. However, it is less likely that the observed negative effect on the organic-cemented agglutinated species resulted from a toxic response to Cr and Mn at the outfall station, considering their low concentrations (below the ERM- effects range median) in the sediments of our studied area compare to [57] (Table 2). It is, therefore, more reasonable to conclude that these species are more affected by the elevated salinity, and could be highly sensitive bioindicators of elevated salinities due to the lack of a dense mineralized protective shell. These species are less common in energetic sandy shallower environments due to their delicate shells, which are not preserved in the sediments. However, they are well known from silty sediments at a 36 m water depth with a high organic matter content near the Palmachim sewage outlet (southeastern Mediterranean Sea) [54,58].

## Summary and conclusions

1. Seasonal changes in benthic foraminiferal assemblages were monitored at three desalination facility sites (Ashkelon, Sorek, and Hadera) along the Mediterranean coast of Israel during 2016–2017. Two of the study sites, Ashkelon and Hadera, are very shallow, and mixing with the cooling water of adjacent power plants occurs at these sites operating since 2005 and 2009, respectively. The outcome is the local elevation of water temperatures in addition to the brine discharged. On the other hand, a diffuser system without cooling water operates at the Sorek site since 2013, allowing us to assess the effect of brine alone on the shallow-water ecosystem, without the added parameter of elevated temperatures.
2. All sites exhibited significant differences between the outfall and the control stations, indicating a robust synergic effect of elevated brine levels and temperature (i.e., Hadera and Ashkelon) as well as an independent influence of brine (Sorek).
3. The most noticeable effects of the brine and temperatures were the decreases in abundance and species richness observed between most of the samples at the outfall and the control stations from the same water depth.
4. The Sorek site facilitated an isolated evaluation of the impact of elevated salinity on the foraminiferal assemblages. The most noticeable negative response to brine discharge was observed among different lineages of organic-cemented agglutinated species. Their similar responses indicated mutual sensitivity of this shell-type strategy to an increase in salinity above ambient levels.
5. This study demonstrates that an increase of 2–3 salinity units caused by desalination plants has a minor, local effect on calcareous benthic foraminiferal assemblages and a more prominent impact on organic-cemented agglutinated species. Therefore, to ensure a minor effect on the local benthic ecosystem, it is pertinent to maintain low levels of brine while the industry continues to evolve.

## Supporting information

S1 Table Summarizing table of all foraminifera data. Numerical abundances (number of individuals per gram dry sediment > 63 μm), the total number of specimens counted, and the number of species, of each site, station, season, and replicate.
(XLSX)

S2 Tables Statistical tests results. STATISTICA 10 software output.
**Table A in S2 Tables.** Two-way ANOVA is comparing salinity measurements of the three studied sites (Ashkelon, Sorek, and Hadera), and the stations (outfall and control) for each site.
**Table B in S2 Tables.** Tukey HSD post-hoc test demonstrating the salinity differences between the outfall to the control of the three sites. Stars indicate homogenous groups.
**Table C in S2 Tables.** One-way ANOVA is comparing salinity measurements of the outfall and control stations at Ashkelon.
**Table D in S2 Tables.** A non-parametric, Mann-Whitney U test comparing salinity measurements of the outfall and control stations at Hadera. This test was performed because the ANOVA Assumption was violated.
**Table E in S2 Tables.** One-way ANOVA is comparing salinity measurements of the outfall and control stations at Sorek.
(PDF)

S1 Fig Two-way ANOVA test results and graphs. Demonstrating the differences of Foraminifera total abundances (top) and species richness (bottom), between the outfall to the control of each site.
(PDF)
