# Supplementary material for: The effect of long-term brine discharge from desalination plants on benthic foraminifera
Source: PLoS One. 2020 Jan 14;15(1):e0227589. doi: 10.1371/journal.pone.0227589 (PMC6959559; doi:10.1371/journal.pone.0227589)
Supplement: S2 Tables — STATISTICA 10 software output. Table A in S2 Tables. Two-way ANOVA is comparing salinity measurements of the three studied sites (Ashkelon, Sorek, and Hadera), and the stations (outfall and control) for each site. Table B in S2 Tables. Tukey HSD post-hoc test demonstrating the salinity differences between the outfall to the control of the three sites. Stars indicate homogenous groups. Table C in S2 Tables. One-way ANOVA is comparing salinity measurements of the outfall and control stations at Ashkelon. Table D in S2 Tables. A non-parametric, Mann-Whitney U test comparing salinity measurements of the outfall and control stations at Hadera. This test was performed because the ANOVA Assumption was violated. Table E in S2 Tables. One-way ANOVA is comparing salinity measurements of the outfall and control stations at Sorek. (PDF) [file pone.0227589.s002.pdf]

S2 Tables. Statistical tests results. STATISTICA 10 software output.

Table A in S2: Two-way ANOVA is comparing salinity measurements of the three studied sites (Ashkelon, Sorek, and Hadera), and the stations (outfall and control) for each site.

Univariate Tests of Significance for salinity (Spreadsheet1)<br>Sigma-restricted parameterization<br>Effective hypothesis decomposition; Std. Error of Estimate: .8824158					
	SS	Degr. of	MS	F	p
Intercept	39530.25	1	39530.25	50767.17	0.000000
station	25.62	1	25.62	32.91	0.000019
site	5.98	2	2.99	3.84	0.040779
station*site	4.23	2	2.11	2.71	0.093261
Error	14.02	18	0.78		

Table B in S2: Tukey HSD post-hoc test demonstrating the salinity differences between the outfall to the control of the three sites. Stars indicate homogenous groups.

Tukey HSD test; variable salinity (Spreadsheet1)<br>Homogenous Groups, alpha = .05000<br>Error: Between MSE = .77866, df = 18.000						
	station	site	salinity	1	2	3
4	control	Ashkelon	39.71843	****	****	
1	Outfall	Ashkelon	42.82000			****
5	control	Hadera	39.57000	****	****	
2	Outfall	Hadera	40.61571	****	****	
6	control	Sorek	39.36508	****		
3	Outfall	Sorek	41.41720		****	****

Table C in S2: One-way ANOVA is comparing salinity measurements of the outfall and control stations at Ashkelon.

Univariate Tests of Significance for salinity (Spreadsheet6)<br>Sigma-restricted parameterization<br>Effective hypothesis decomposition; Std. Error of Estimate: 1.134276					
	SS	Degr. of	MS	F	p
Intercept	13625.18	1	13625.18	10590.22	0.000000
station	19.24	1	19.24	14.95	0.008294
Error	7.72	6	1.29		

Table D in S2: A non-parametric, Mann-Whitney U test comparing salinity measurements of the outfall and control stations at Hadera. This test was performed because the ANOVA Assumption was violated.

Mann-Whitney U Test (Spreadsheet14)<br>By variable station<br>Marked tests are significant at p <.05000										
	Rank Sum	Rank Sum	U	Z	p-value	Z	p-value	Valid N	Valid N	2*1sided
salinity	22.00000	14.00000	4.000000	1.010363	0.312322	1.010363	0.312322	4	4	0.342857

Table E in S2: One-way ANOVA is comparing salinity measurements of the outfall and control stations at Sorek.

Univariate Tests of Significance for salinity (Spreadsheet18)<br>Sigma-restricted parameterization<br>Effective hypothesis decomposition					
	SS	Degr. of	MS	F	p
Intercept	13051.55	1	13051.55	19371.66	0.000000
station	8.42	1	8.42	12.50	0.012283
Error	4.04	6	0.67		
