# Supplementary material for: The effect of long-term brine discharge from desalination plants on benthic foraminifera
Source: PLoS One. 2020 Jan 14;15(1):e0227589. doi: 10.1371/journal.pone.0227589 (PMC6959559; doi:10.1371/journal.pone.0227589)

S1 Figure. Two-way ANOVA test results and graphs. Demonstrating the differences of Foraminifera total abundances (top) and species richness (bottom), between the outfall to the control of each site.

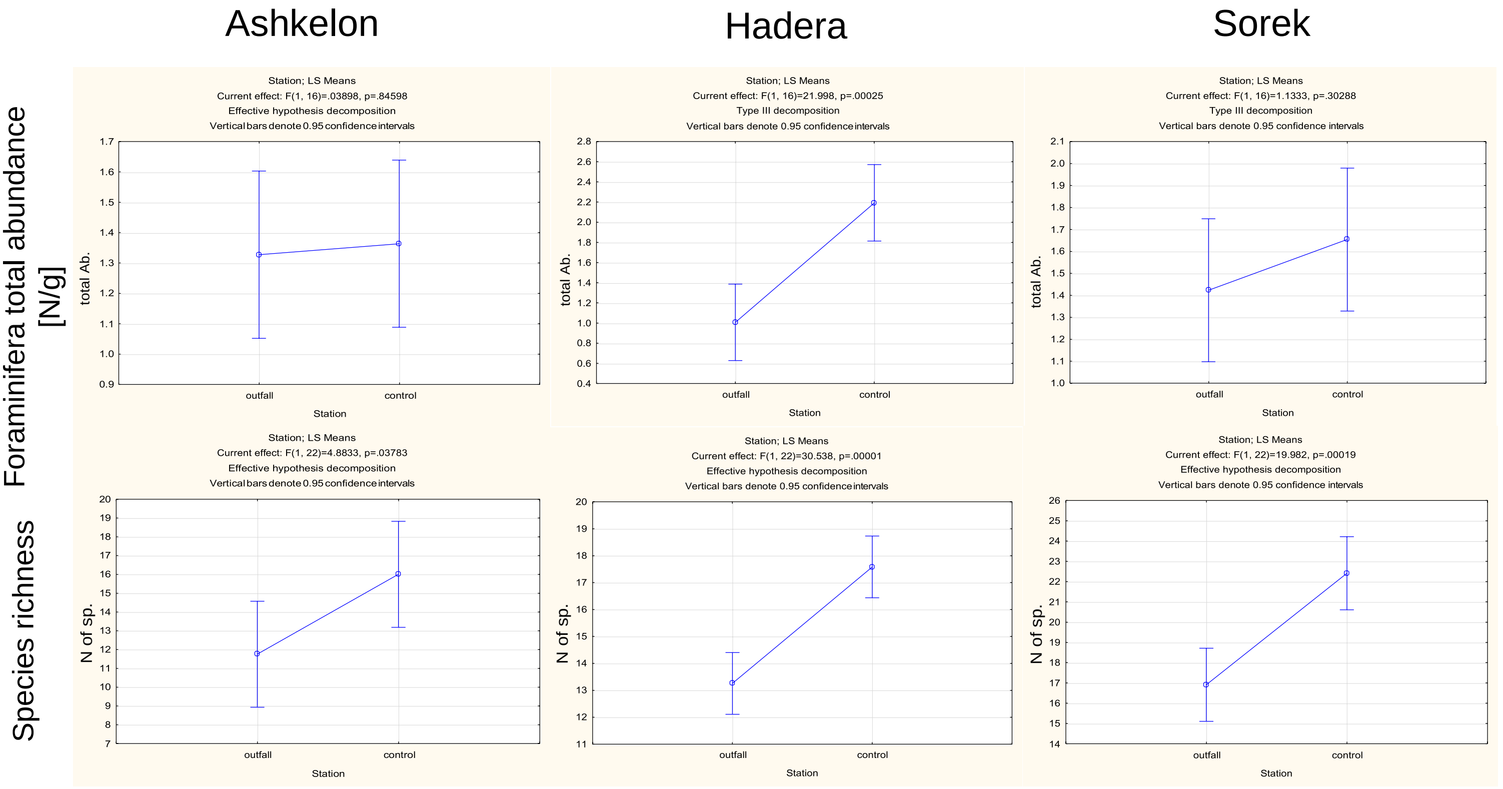

Supplement: S1 Fig — Demonstrating the differences of Foraminifera total abundances (top) and species richness (bottom), between the outfall to the control of each site. (PDF) [file pone.0227589.s003.pdf]
